# Costs and benefits of influenza vaccination: more evidence, same challenges

**DOI:** 10.1186/1471-2458-14-818

**Published:** 2014-08-08

**Authors:** Bruno Christian Ciancio, Giovanni Rezza

**Affiliations:** European Centre for Disease Prevention and Control (ECDC), Stockholm, Sweden; Department of Infectious, Parasitic and Immunomediated Diseases, Istituto Superiore di Sanità, Roma, Italy

**Keywords:** Influenza burden, Vaccine effectiveness, Cost

## Abstract

Seasonal influenza vaccination coverage in most EU/EEA remains suboptimal. Providers’ and users’ confidence in influenza vaccines is undermined by reports of moderate to low vaccine effectiveness and by the lack of solid evidence on disease burden. A study from Preaud and co. indicates that even with current levels of vaccine effectiveness, increasing vaccination coverage would significantly reduce disease burden and health cost. The results of the study should be interpreted cautiously because some of the assumptions are not generalizable or are imprecise, especially those on vaccine coverage, disease burden and health cost. Increasing vaccination coverage in EU/EEA countries is very challenging. Multifaceted approaches and country specific strategies are needed to address vaccine hesitancy in health care workers and in the population, and to manage organisational and financial obstacles. One key element for increasing vaccination coverage is the development of better influenza vaccines, e.g. vaccines that are more effective, provide longer lasting immunity and do not require annual administration. Vaccine producers should consider this as the highest research priority in the field of influenza vaccine development.

## Background

Influenza viruses are characterized by continuous antigenic evolutions; thus, annual revaccination is needed to maintain an adequate level of immunological protection among those at risk of severe illness. In order to plan vaccination programmes, updated information on disease burden and associated costs, vaccination coverage, reasons for vaccine hesitancy and low coverage at population level, and risk/benefit analysis of vaccination is needed. At EU/EEA level, such information is often difficult to obtain, due to heterogeneity among Member States in the availability of resources, quality of surveillance systems, and comprehensive vaccination registries. Despite the 2009 European Council recommendations [1], seasonal influenza vaccination coverage in EU/EEA is mostly suboptimal, with some countries being very far from the target of 75% coverage in older individuals and in other target groups [2].

In this issue of BMC Public Health, Preaud and colleagues from vaccine producer firms report estimates of the annual impact of influenza epidemics in terms of morbidity/mortality and offset costs for 27 EU/EEA countries [3]. The study provides insights into current Influenza vaccine coverage gaps and possible benefits of increasing coverage levels. The authors estimated that additional 57.4 million individuals should be vaccinated every year to achieve 75% coverage in the target groups: between 1.6 and 1.7 million influenza cases would be prevented each year, as well as more than half million general practitioners visits, 23,800-31,400 hospitalisations, 9,800-14,300 deaths, and almost 1 million lost days of work. As expected, the highest impact in terms of averted hospitalisations and deaths would be among individuals older than 64 years. Overall, between € 190 million and € 226 million yearly would be saved.

Due to the lack of country specific data for many EU/EEA countries included, the results of the study were based on a number of assumptions and extrapolations.

Annual influenza attack rates by risk group were derived from the placebo arms of randomised controlled trials, with overrepresentation of healthy adults and children. Country specific data on disease burden by risk group and offset costs were available from few countries and extrapolated to the others based on geographical proximity and similarities of health care systems. Influenza related hospitalisations and mortality data are also very scanty, and estimating the burden of disease as the excess of all-cause hospitalisations and deaths may have led to an overestimation, whereas using Eurostat case-specific events may have led to an underestimation of the burden, because very few influenza deaths and hospitalisations are recorded as such. In addition the study tends to underestimate the disease burden in children, pregnant women and young adults with underlying conditions. Using disability-adjusted life years (DALYs) instead of morbidity/mortality, would have better accounted for premature mortality (years of life lost) and long term sequelae in these groups. Vaccine coverage figures were obtained from published sources, when available, and extrapolated for all countries and risk groups for which information was not available. Vaccination coverage figures in Europe are quite reliable for individuals older than 64 years, but much less for other target groups. Only few countries have comprehensive vaccination registries and few *ad hoc* studies are available to provide reliable figures. Thus, the estimation of the vaccine gap is likely to be imprecise.

Analyses were performed separately for vaccine effectiveness, vaccine efficacy and their upper and lower 95% confidence intervals (CI), and by age group and presence of underlying conditions. Overall vaccine effectiveness estimates were obtained from a single US study performed during the 2010/11 influenza season where co-circulation of influenza A (H1N1), A(H3N2) and B was observed [4]. Estimates ranged from 56% (95% CI: 31 to 74) among children 6 months-2 years of age to 36% (95% CI: 22 to 66) among subjects ≥65 years. Ninety five percent CI were obtained from two European studies performed during the same [5] and the following season [6] when there was dominance of influenza A(H1N1) and A (H3N2), respectively. Vaccine efficacy values and their 95% CI were obtained from Cochrane literature reviews. Although these estimates are based on valid studies, vaccine effectiveness estimates are season- and strain-specific (due to factors such as variations in the vaccine/virus match and the time between vaccination and exposure), thus affecting the generalizability of the figures on averted cases and costs.

Despite these limitations, we agree with the conclusions of the study that even with suboptimal vaccine effectiveness, the benefits of increasing vaccination coverage would be enormous in terms of averted morbidity, mortality and cost. It is therefore important to mention some of the main reasons for low vaccine coverage in EU/EEA countries:

1. Vaccine hesitancy in health care workers (HCW) and in the population. According to the annual surveys conducted by ECDC through the VENICE network, vaccination coverage among HCWs is very low in EU and EEA countries [2]. The motivation of HCW plays an important role in the effectiveness of influenza vaccination programmes. Doubts about vaccine effectiveness and/or safety [7–10] and disease severity [9, 11] are the main reasons for HCW refusing seasonal vaccination. HCW with these concerns are also less likely to recommend vaccination to their patients.
2. Lack of confidence in vaccine effectiveness and safety. In recent years, also due to limited availability of high quality evidence [12] and controversial publications on vaccine effectiveness [13, 14], Europe has experienced a surge of doubts about effectiveness of influenza vaccines. Reliable systems should be further implemented to monitor vaccine effectiveness and safety [15–17], in order to build trust in influenza vaccines among HCW and the general population.
3. Complex vaccine recommendations. Higher vaccination coverage among elderly compared to other target groups are also explained by a clear age cut-off defining this group. Among other reasons (i.e., to reduce virus circulation in the community), these considerations have led the US Centers for Disease Control and Prevention to recommend universal vaccination of the population older than 5 months of age in the US [18]. This is also a way to indirectly reach those with underlying conditions in some population groups [19].
4. Short vaccine delivery window. Most influenza vaccination campaigns aim at administering the seasonal vaccine to the highest number of eligible subjects during a short time window preceding the start of the flu season. To overcome organisational challenges, vaccination should also be encouraged during the entire influenza season.
5. Lack of resources. In a time of severe financial constraints and in the absence of strong data on influenza burden and effectiveness of vaccination, it is difficult for many EU/EEA countries to prioritise influenza prevention over other perceived more pressing health issues.

In conclusion, seasonal influenza vaccination is the single most effective protective measure against influenza. In order to maximise the effectiveness of influenza vaccination programmes vaccination coverage should be monitored more effectively in EU/EEA countries using vaccination registries or other administrative methods in combination with periodic population surveys. This may facilitate identification of the groups where vaccine coverage is low and the reasons for being unvaccinated, in order to inform targeted interventions to increase coverage. Surveillance systems should be strengthened to provide accurate estimates of disease burden by age and risk group. Vaccine effectiveness and safety monitoring should be ensured at EU/EEA level, rising population and HCW trust on recommended interventions. Finally, research on immunological correlates of protection is needed to stimulate development of more effective vaccines (e.g. wider/universal strain coverage and longer lasting immunity) and to increase their acceptance by doctors and patients. This research is expensive and unaffordable for most public health agencies and requires stronger financial commitments from the private sector. Vaccine producers should consider this as the highest research priority in the field of influenza vaccine development [20].
